# Ultrasound-assisted intravesical botulinum toxin A delivery attenuates acetic acid—induced bladder hyperactivity in rats

**DOI:** 10.3389/fphar.2023.1214145

**Published:** 2023-07-24

**Authors:** Qinggang Liu, Yi Gao, Huiling Cong, Limin Liao

**Affiliations:** ^1^ Cheeloo College of Medicine, Shandong University, Jinan, Shandong, China; ^2^ Department of Urology, China Rehabilitation Research Center, Beijing, China; ^3^ School of Health and Life Sciences, University of Health and Rehabilitation Sciences, Qingdao, Shandong, China; ^4^ China Rehabilitation Science Institute, Beijing, China; ^5^ Beijing Key Laboratory of Neural Injury and Rehabilitation, Beijing, China; ^6^ School of Rehabilitation, Capital Medical University, Beijing, China

**Keywords:** ultrasound, microbubbles, botulinum toxin A, intravesical delivery, bladder hyperactivity

## Abstract

**Background:** Intradetrusor injection of botulinum toxin A (BTX-A) is an effective treatment for overactive bladder (OAB). However, the occurrence of adverse events associated with BTX-A injection therapy hinders its acceptance among patients and its clinical promotion. Intravesical instillation of BTX-A offers a promising alternative to injection therapy for treating OAB. Nevertheless, due to the presence of the bladder permeability barrier (BPB) and the high molecular weight of BTX-A, direct instillation is unable to penetrate the bladder urothelium.

**Purpose:** This study aims to investigate the safety and feasibility of ultrasound-assisted intravesical delivery of BTX-A and its potential benefits in a rat model of bladder hyperactivity induced by acetic acid instillation.

**Methods:** Hengli BTX-A and microbubbles (MB) were mixed and prepared as a novel complex. The size distribution and zeta potentials of the complex were measured. On day 1, rats’ bladders were instilled with 1 mL of saline, BTX-A (20 U in 1 mL), MB, or MB-BTX-A (20 U in 1 mL) complex with or without ultrasound (US) exposure (1 MHz, 1.5 W/cm^2^, 50% duty cycle, sonication for 10 s with a 10-s pause for a total of 10 min). The instillations were maintained for 30 min. After 7 days, cystometry was performed by filling the bladder with saline and 0.3% acetic acid (AA). Bladders were collected, weighed, and processed for immunoblotting, enzyme-linked immunosorbent assay (ELISA), histologic, and immunofluorescence analyses. Expression and distribution of SNAP-25 and SNAP-23 were assessed using Western blot and immunofluorescence. Calcitonin gene-related peptide (CGRP) in the bladder was detected using ELISA.

**Results:** Intercontraction intervals (ICI) decreased by 72.99%, 76.16%, and 73.96% in rats pretreated with saline, BTX-A, and US + MB, respectively. However, rats treated with US + MB + BTX-A showed a significantly reduced response to AA instillation (57.31% decrease in ICI) without affecting amplitude, baseline pressure, or threshold pressure. Rats treated with US + MB + BTX-A exhibited increased cleavage of SNAP-25 and CGRP expression compared to the control group.

**Conclusion:** Ultrasound-assisted intravesical delivery of BTX-A, with the assistance of MB cavitation, led to cleavage of SNAP-25, inhibition of calcitonin gene-related peptide release from afferent nerve terminals, and amelioration of acetic acid-induced bladder hyperactivity. These results support ultrasound-assisted intravesical delivery as an efficient non-injection method for administering BTX-A.

## 1 Introduction

Botulinum toxin A (BTX-A) has been utilized for over 30 years as a treatment for lower urinary tract disorders (LUTDs). The recommended dosages for overactive bladder (OAB) and neurogenic detrusor overactivity (NDO) are 100 and 200 U, respectively (Jiang et al., 2015). BTX-A exerts its effects by proteolyzing synaptosomal-associated protein 25 (SNAP-25), an essential component of the soluble N-ethylmaleimide-sensitive factor attachment protein receptor (SNARE) complex responsible for neurotransmitter-containing vesicle exocytosis in the bladder. Recent studies have demonstrated that BTX-A injections can diminish bladder sensory nerve function by reducing the expression of various receptors on afferent nerve fibers, such as ATP receptors P2X3 and transient receptor potential vanilloid subfamily-1 (TRPV1) (Chuang et al., 2010). Additionally, intravesical BTX-A injections in rodent bladders have been shown to reduce SNAP-23 levels and inhibit ATP and neurotrophin release from the urothelium while increasing nitric oxide levels (Ibrahim et al., 2022).

Due to its 150 kDa molecular weight, BTX-A cannot penetrate the submucosal nerve plexus when administered in saline. In clinical practice, BTX is directly injected through the urothelium. However, BTX-A injection therapy is associated with common and bothersome adverse events, including drug leakage, uneven distribution, hematuria, pain, and infection risk (Kuo, 2022). Therefore, there is an urgent need for a safe and effective method of preparing and delivering BTX-A that can replace the conventional direct injection approach.

Ultrasound (US)-triggered microbubbles cavitation, also known as sonoporation, is a minimally invasive and effective drug delivery method. When exposed to US, oscillating and collapsing gas-filled microbubbles (MB) transiently increase cell membrane permeability, allowing for the uptake of extracellular molecules (Chowdhury et al., 2020). Sonoporation has garnered attention for various therapeutic applications, including blood-brain barrier permeabilization (Wang et al., 2022), thermal ablation (Luo et al., 2007), and targeted delivery of drugs or genes via MB (Deprez et al., 2021). Through sonoporation, drugs can be uniformly distributed within the target site and more efficiently absorbed by the body. This approach is particularly applicable to the bladder, which is lined with the bladder permeability barrier (BPB), consisting of tight junctions connecting umbrella cells, densely packed plaques, and glycosaminoglycan (GAG) mucin (GuhaSarkar and Banerjee, 2010). By applying higher-intensity US pulses to this region, the drug can be delivered into the bladder tissue, limiting side effects in other areas. Increasing evidence supports the use of US-assisted intravesical drug delivery for the treatment of urinary tract infections (UTIs) and non-muscle invasive bladder cancer (NMIBC) (Horsley et al., 2019; Sasaki et al., 2022).

In this study, we aimed to investigate whether US-mediated sonoporation could transiently increase urothelium permeability and facilitate BTX-A delivery. We utilized a rat model of acute overactive bladder induced by 0.3% acetic acid (AA) instillation to evaluate the efficacy of this approach in ameliorating hyperactive bladder symptoms. To confirm the delivery effectiveness of BTX-A and assess its pharmacological targets, SNAP-25 and SNAP-23 were investigated.

## 2 Material and methods

### 2.1 Animals

Female Sprague-Dawley (SD) rats, aged 8–10 weeks and weighing 200–220 g, were used in this study. The rats were housed in a temperature-controlled room with free access to food and water. All animal procedures were conducted in compliance with the institutional guidelines for the care and use of laboratory animals and were approved by the Animal Care and Use Committee at Capital Medical University.

### 2.2 Preparation and characterization of microbubbles and botulinum toxin A

BTX-A (Hengli, Lanzhou Institute of Biological Products, China) was dissolved in 5 mL physiological saline to achieve a final concentration of 20 U/mL. Perfluoropropane microbubbles (SunLipo NanoTech, China) were prepared following the manufacturer’s instructions. The MB and BTX-A mixture was obtained by dissolving freeze-dried BTX-A powder in 5 mL of MB solution. This MB and BTX-A mixture was selected for subsequent experiments. The morphology of the microbubbles was examined using a transmission electron microscope (Ht-7700, Hitachi). The size distribution and zeta potential of the microbubbles were measured using a particle size and zeta potential analyzer (Malvern Instruments).

### 2.3 *In vivo* US exposure and drug delivery

To initiate the US exposure and drug delivery process, each rat was anesthetized by intraperitoneal injection of urethane (1.2 g/kg). Abdominal fur was removed. A NU-TEK UT1041 US stimulator (Shenzhen Dongdixin Technology Co., Ltd., Shenzhen, China) with a 1-cm2 transducer was used to position the bladder for subsequent US treatment. The transducer was coupled to the skin in the bladder region using US coupling gel. Local US was applied to the bladder dome using a sonoporator operating at 1 MHz frequency, 1.5 W/cm^2^ intensity, 50% duty cycle, and 10 s of sonication followed by a 10-s pause for a total duration of 10 min. Rats without US exposure were used as control subjects.

After performing control cystometry with saline infusion on day 1, a PE-50 tube was inserted into the bladder through the urethra and secured at the external urethral orifice using a ligature under anesthesia. The bladder was emptied, and then either the MB and BTX-A mixture (1 mL, 20 U BTX-A) or saline (1 mL) was instilled into the bladder via the catheter. Following the instillation, the bladder was exposed to US or sham treatment for 10 min. After the US or sham treatment, the MB and BTX-A mixture or saline remained in the bladder for 30 min until the catheter was removed. Rats were maintained in the physiologic range using a heating lamp to maintain their body temperature until awake.

### 2.4 Cystometry/cystometrogram (CMG)

Rats were anesthetized using intraperitoneal urethane injections (1.2 g/kg). A PE-50 tube was inserted into the bladder through the urethra and connected via a 3-way stopcock to a pressure transducer (MP150, Biopac, CA, United States) and a syringe pump to record intravesical pressure and instill solutions into the bladder. Baseline cystometry was conducted on day 1 by gradually filling the bladder with saline at a rate of 0.08 mL/min to elicit repetitive voiding. Parameters such as pressure amplitude, threshold pressure, baseline pressure, and intercontraction intervals (ICIs) of reflex bladder contractions were measured. An average of 3–5 bladder contractions was recorded in each animal. On day 8, a 0.3% AA solution was infused into the bladder to induce acute bladder hyperactivity after baseline measurements with saline. Subsequently, 3 to 5 bladder contractions were observed and recorded.

### 2.5 Animal model and experimental groups

After conducting control CMGs with saline instillation, rats were assigned to one of four groups based on the intravesical instillation of different solutions and US exposure: (a) instillation of saline for 30 min; (b) instillation of BTX-A (1 mL, 20 U/mL) for 30 min; (c) instillation of MB and US exposure for 10 min, followed by a 30-min maintenance period; (d) instillation of BTX-A (1 mL, 20 U/mL) and the MB mixture, followed by exposure to US for 10 min and a subsequent 30-min maintenance period.

### 2.6 Histology and immunofluorescence staining

On day 8 after the cystometry study, animals were euthanized with isoflurane, and the bladders were harvested. Each bladder was halved for histological, immunofluorescent, and Western blot analyses. For histologic analysis, bladder tissues were fixed in 4% paraformaldehyde for 24–48 h, followed by embedding in paraffin. Four-micrometer sections were stained with hematoxylin and eosin.

Immunofluorescence staining of tissue sections involved blocking 4-μm sections with goat serum, incubating them with primary antibodies overnight at 4°C, and then incubating them with Alexa Fluor 488 Goat Anti-Rabbit IgG (1:200, Yeasen, China) or Alexa Fluor 594 Goat Anti-Rabbit IgG (1:200, Yeasen, China) for 1 h at 37°C. After DAPI staining, fluorescent images were captured using a fluorescence microscope.

### 2.7 Western blot analysis

For Western blot analysis, the rat bladder samples were processed according to the standard protocol (Beyotime, Shanghai, China). Total protein was extracted and quantified using the bicinchoninic acid (BCA) protein assay method. Approximately 40 μg of protein from each sample was separated on 8%, 10%, or 15% SDS-PAGE gels and transferred to polyvinylidene difluoride (PVDF) membranes. The membranes were then blocked with defatted milk and incubated overnight at +4°C with specific primary antibodies, including rabbit anti-E-Cadherin polyclonal antibody (1:5,000, Proteintech, United States), rabbit anti-ZO-1 polyclonal antibody (1:5,000, Proteintech, United States), rabbit anti-SNAP-23 polyclonal antibody (1:2000, Abcam, United States), mouse anti-cleaved and uncleaved SNAP-25 monoclonal antibody (1:100, GeneTex, United States), and rabbit anti-GAPDH polyclonal antibody (1:5,000, Proteintech, United States). Subsequently, the membranes were incubated with horseradish peroxidase-conjugated secondary antibody (1:3,000, Proteintech, United States) at room temperature for 1 h. Protein bands were visualized and analyzed using the Bio-Rad ChemiDoc Imagers System.

### 2.8 Enzyme-linked immunosorbent assay

CGRP levels in bladder tissue were measured using an ELISA kit (CSB-E08211r, Cusabio, United States) following the manufacturer’s instructions. Bladder tissues (80 mg) were rinsed in 800 μL of PBS. Total proteins were extracted after 2 freeze-thaw cycles with liquid nitrogen to disrupt cell membranes, and the homogenates were centrifuged for 10 min at 12,000 g at 4°C to collect the supernatants. Then, 100 μL of standard or sample was added per well, and the plate was incubated at 37°C for 2 h. After incubation, the liquid was removed, and a 1x biotin-antibody solution was added to each well and incubated for 1 h at 37°C. Each well was washed three times with an aspirate before adding 100 μL of HRP-avidin solution, which was incubated for 1 h at 37°C. Following five washes, the substrate solution was added to each well and incubated for 15–30 min at 37°C. Finally, 50 μL of stop solution was added to each well, and the optical density of each well was determined within 5 min using a microplate reader set at 450 nm.

### 2.9 Statistical analysis

Statistical analysis was performed using the Statistical Package for Social Science (SPSS, version 25) and GraphPad Prism 9 software. Data were expressed as mean ± SD. Differences between groups were assessed using one-way analysis of variance (ANOVA). A *p*-value of less than 0.05 was considered statistically significant.

## 3 Results

### 3.1 Characterization of microbubbles and ultrasound exposure

**FIGURE 1:**
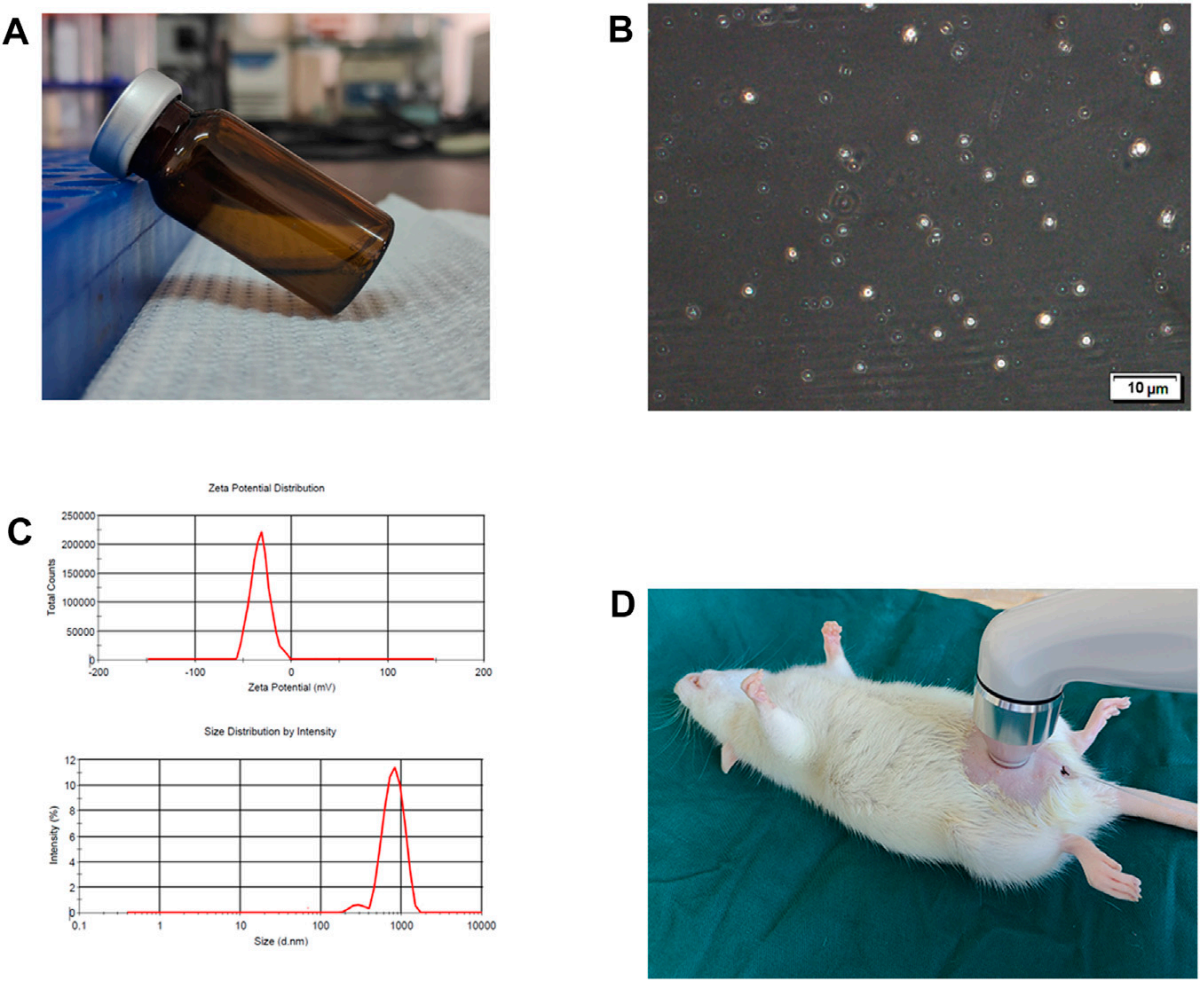
Characteristics of microbubbles and ultrasound exposure. **(A)** Microbubbles (MB) were prepared according to manufacturer’s instructions. **(B)** Representative transmission electron microscope image of MB. **(C)** Hydrodynamic diameter distribution and zeta potential for MB. **(D)** Diagram of ultrasound (US) exposure.

The microbubbles (MB) utilized in the study were prepared following the manufacturer’s instructions, and their characteristics were examined. (Figure 1A). A well-defined spherical shape of MB was observed in transmission electron microscope (TEM) images (Figure 1B). The dynamic light scattering (DLS) analysis demonstrated an average hydrodynamic diameter of 874.3 nm for the MB (Figure 1C). The MB exhibited an average zeta potential of −26.2 mV (Figure 1C). To enable the delivery of MB and BTX-A mixture, a transducer/sonoporator was applied externally to the entire bladder within the peritoneal cavity (Figure 1D).

### 3.2 Histological response to saline or microbubbles instillation with or without ultrasound

**FIGURE 2:**
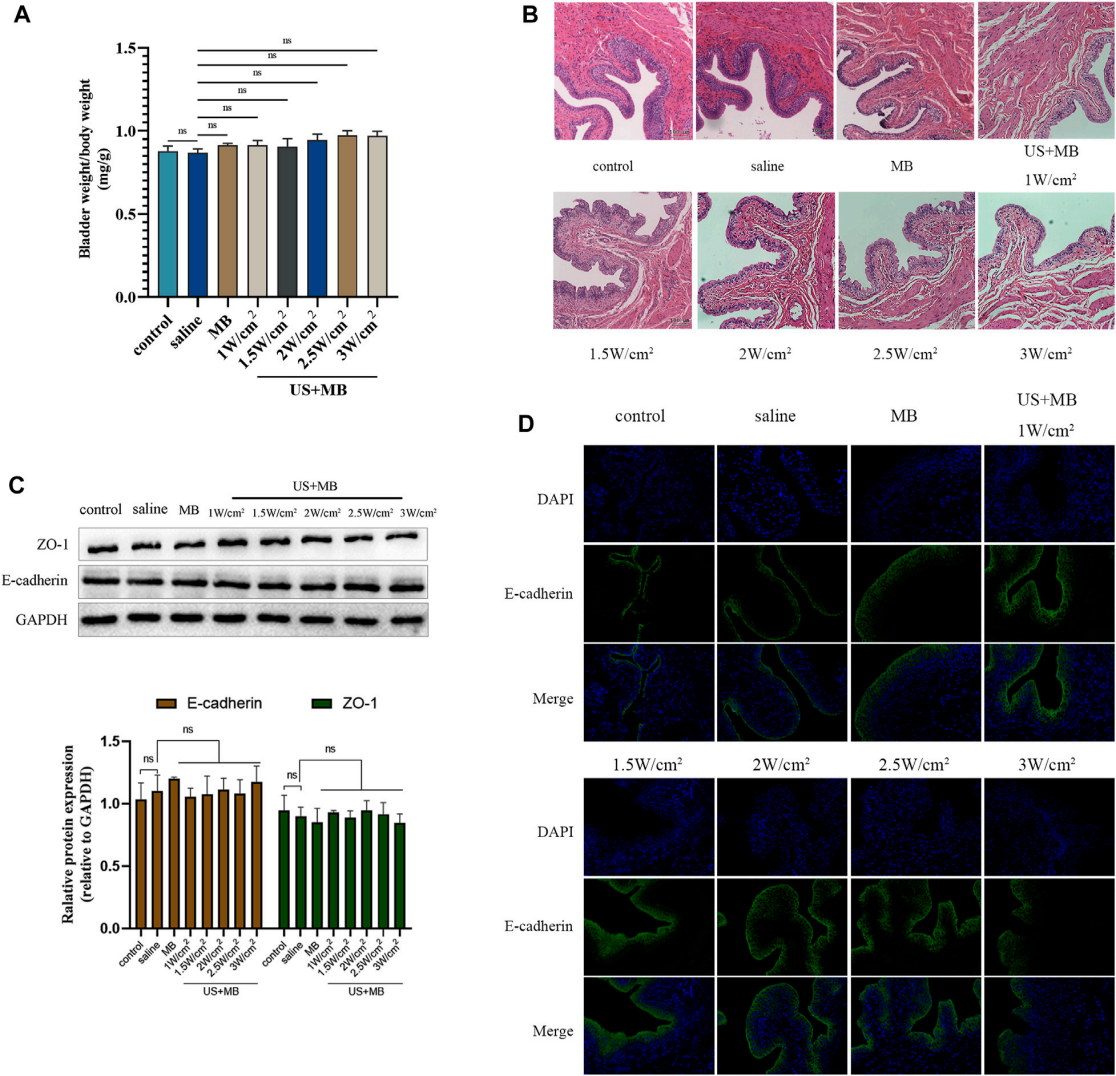
Biocompatibility to bladder tissue of microbubbles with and without ultrasound *in vivo*. **(A)** Sprague-Dawley rats underwent intravesical instillation of saline or microbubbles with and without ultrasound 30 min. Bladder and body weights in each group. Bars represent mean ± SD. **(B)** Hematoxylin and eosin staining of bladders samples. **(C)** Expression of ZO-1 and E-cadherin analyzed using Western blot. Bars represent mean ± SD. **(D)** Immunofluorescence staining of E-cadherin protein in bladder samples.

There were no significant differences in bladder weight between the groups that received saline or microbubbles (MB) instillation, with or without ultrasound treatment (Figure 2A). The US + MB groups, regardless of ultrasound intensity, showed mild submucosal edema and thickening of the submucosal structure. There were no significant differences in submucosal edema and submucosal structure changes among the different ultrasound intensity groups combined with MB (Figure 2B).

### 3.3 Effects of microbubbles instillation with or without ultrasound on E-cadherin and ZO-1 expression and distribution

Bladder tissue was harvested 7 days after saline or MB instillation, with or without ultrasound, for Western blot and immunofluorescent analysis of E-cadherin and ZO-1 protein expression and distribution. No significant differences were observed between the groups in the expression of E-cadherin or ZO-1 (Figure 2C). The immunofluorescent assay revealed consistent and intact distribution of E-cadherin among the groups (Figure 2D).

### 3.4 Cystometry response to saline, microbubbles, and BTX-A with or without ultrasound pretreatment

**FIGURE 3:**
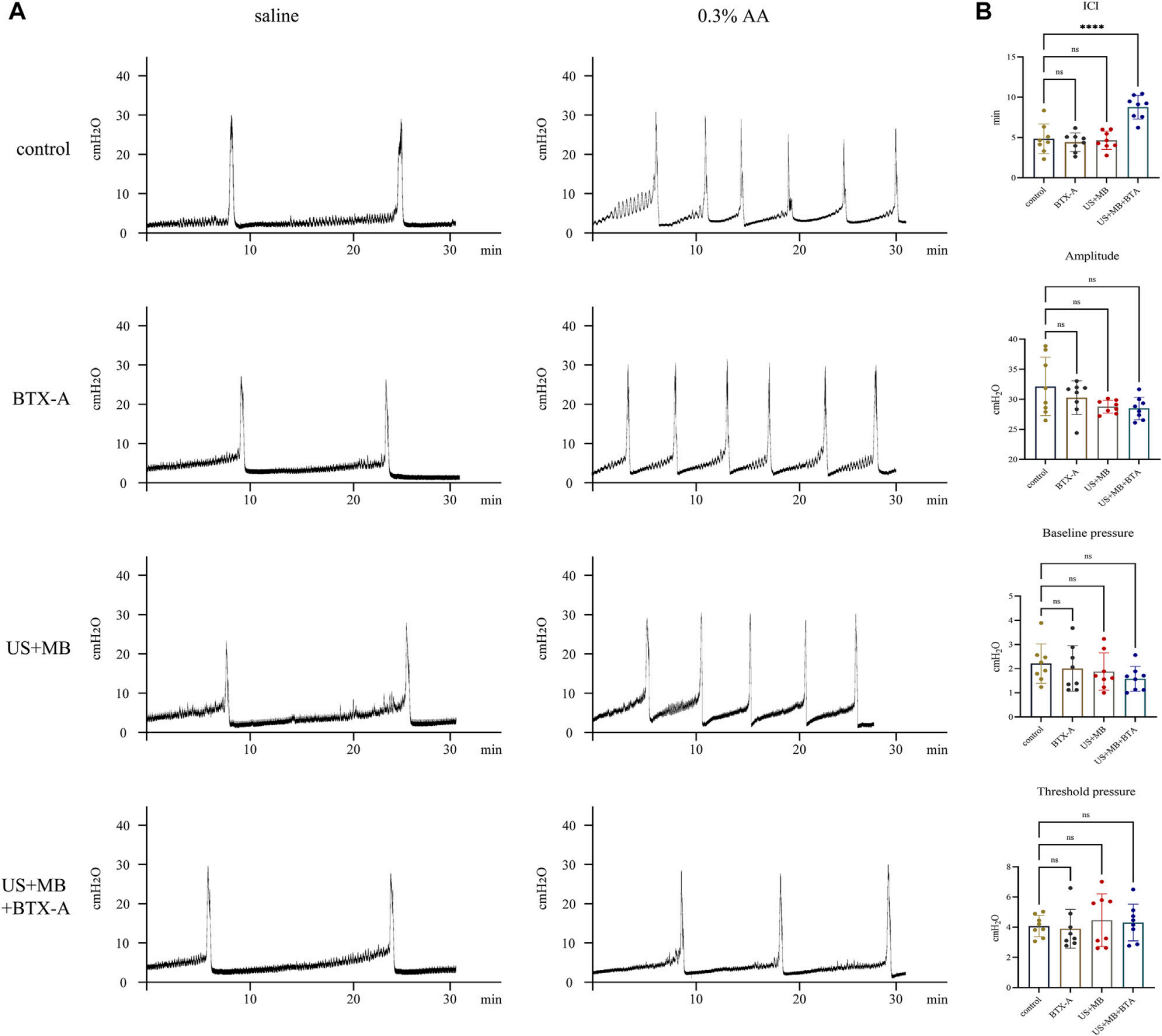
Representative cystometrogram tracings after saline, botulinum toxin A, and/or microbubbles instillation with and without ultrasound. **(A)** Representative tracings of intraurethral cystometrograms (CMG) were recorded in urethane-anesthetized rats. CMG was performed in saline, botulinum toxin A (BTX-A)-, US + MB-, and US + MB + BTX-A–pretreated rats. **(B)** Bar plots represent the CMG parameters of amplitude, pressure threshold, pressure baseline, and intercontraction intervals between groups. Bars represent mean ± SD. ****, *p* < 0.0001 by one-way analysis of variance.

Compared to the control group, none of the treated groups exhibited significant differences in baseline CMG parameters prior to AA installation, which resulted in a decrease in ICI across all four groups (Figures 3A, B). The decreased ICI ratios of AA/saline instillation were 72.99% ± 11.58%, 76.16% ± 6.55%, 73.96% ± 7.41%, and 57.31% ± 8.88% for the control, BTX-A, US + MB, and US + MB + BTX-A groups, respectively. The combination of BTX-A pretreatment with US + MB effectively inhibited AA-induced bladder hyperactivity (Table 1).

**TABLE 1:** Cystometric parameters in rats from different groups during saline or 0.3% AA instillation.

	Saline				0.3% AA			
	Control	BTX-A	US + MB	US + MB + BTX-A	Control	BTX-A	US + MB	US + MB + BTX-A
Intercontractile interval (mins)	18.20 ± 2.56	18.74 ± 2.01	18.13 ± 1.84	20.78 ± 1.87	4.84 ± 1.85	4.42 ± 1.15	4.66 ± 1.13	8.76 ± 1.47****
Amplitude (cmH_2_O)	27.30 ± 3.10	26.79 ± 4.12	28.43 ± 3.05	26.33 ± 2.57	32.13 ± 4.86	30.25 ± 2.8	28.75 ± 1.08	27.82 ± 2.07
Baseline pressure (cmH_2_O)	2.91 ± 0.87	2.39 ± 0.72	2.47 ± 0.58	2.30 ± 0.36	2.21 ± 0.81	2.01 ± 0.94	1.88 ± 0.77	1.58 ± 0.51
Threshold pressure (cmH_2_O)	4.87 ± 0.96	4.67 ± 0.91	4.69 ± 0.43	4.52 ± 0.91	4.08 ± 0.71	3.89 ± 1.28	4.46 ± 1.74	4.31 ± 1.21

****, significant difference observed at *p* < 0.0001 when comparing the US + MB + BTX-A, group to the control group. Statistical analysis was conducted using one-way analysis of variance (ANOVA) with the Bonferroni post-test applied when applicable. A significance level of *p* < 0.05 was considered statistically significant.

### 3.5 Expression of SNAP23, SNAP-25, and CGRP in the bladder

**FIGURE 4:**
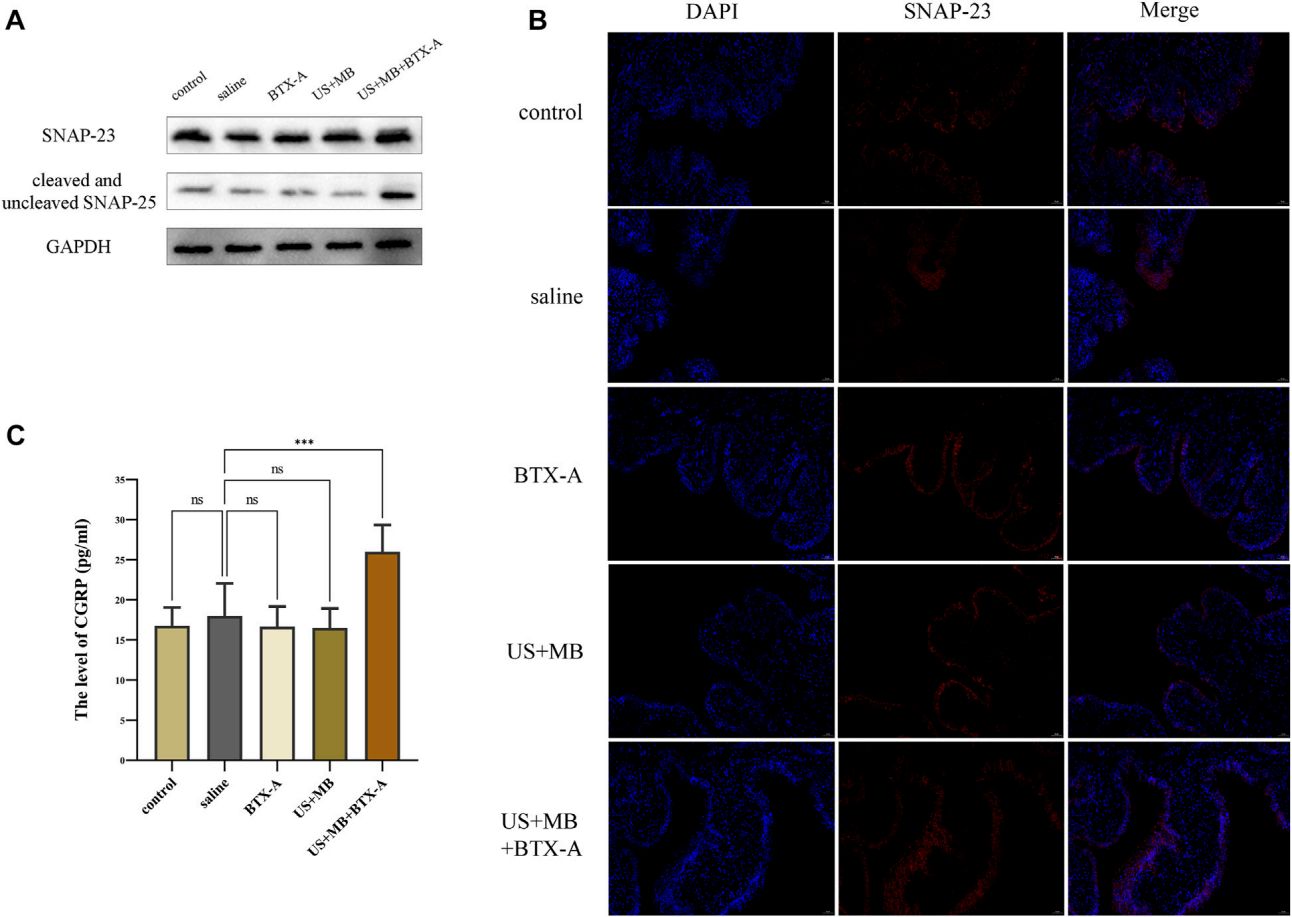
SNAP 23, cleaved and uncleaved SNAP-25, and CGRP expression in bladder tissue. **(A)** After cystometry, rats in each group were euthanized, and bladders were collected for subsequent Western blotting and immunofluorescence staining. Proteins levels of SNAP 23, cleaved and uncleaved SNAP-25 in bladder tissues of each group. **(B)** Representative immunofluorescence staining of SNAP 23 in bladder samples with or without ultrasound or microbubbles treatment. Scale bar = 50 μm. **(C)** Quantitative level of CGRP expression in bladder homogenates between groups using ELISA. Bars represent mean ± SD. **, *p* < 0.01; ***, *p* < 0.001; ****, *p* < 0.0001 by one-way analysis of variance.

Immunostaining of SNAP-23 revealed predominant localization in the apical layer of the bladder urothelium in the control group, which was not significantly different from the BTX-A, US + MB, or US + MB + BTX-A groups (Figure 4A). There were no notable differences in SNAP-23 expression between the groups. However, in the US + MB + BTX-A group, the total expression of SNAP-25 (including cleaved and uncleaved forms) exhibited a significant increase compared to the other groups. Conversely, no significant differences were observed in the control, BTX-A, and US + MB-treated groups (Figures 4A, B). ELISA results demonstrated a 15% increase (*p* < 0.05) in CGRP protein levels in the US + MB + BTX-A group compared to the control group. However, there were no significant differences detected in the BTX-A and US + MB groups compared to the control group (Figure 4C).

## 4 Discussion

The main finding of our study was the suppression of AA-induced bladder hyperactivity through US-triggered MB cavitation-assisted intravesical delivery of BTX-A. This therapeutic effect was not observed in the US + MB or BTX-A saline solution pretreatment groups. Additionally, the US + MB + BTX-A-treated group did not show any changes in bladder contraction amplitude, urinary retention, or overflow incontinence. Interestingly, the expression of cleaved SNAP-25 and CGRP in the US + MB + BTX-A pretreatment group was significantly higher compared to the US + MB and BTX-A saline solution pretreatment group.

Intravesical drug delivery holds promise for the treatment of urinary system diseases. Compared to BTX-A injection, instilling BTX-A directly into the bladder simplifies treatment by eliminating the need for anesthesia and cystoscopy, thereby reducing costs for patients with refractory overactive bladder (Chen et al., 2020; Abrar et al., 2021). However, the effectiveness of intravesical BTX-A delivery is hindered by various factors. The urothelium’s barrier restricts the passive diffusion of BTX-A due to its large molecular weight (150 KDa). Additionally, the intact bladder permeability barrier prevents BTX-A from adhering to and penetrating the bladder, while urine dilution hampers BTX-A localization within the bladder (Tyagi et al., 2006). Various attempts have been made to enhance BTX-A efficacy using physical or chemical methods such as electromotive force, protamine sulfate, chitosan, and dimethylsulphoxide, but these approaches have yet to be optimized for widespread use (GuhaSarkar and Banerjee, 2010).

There is growing interest in using US-activated MB as an intravesical delivery method for bladder diseases. Gas bubbles stabilized by a polymer or surfactant coating have been clinically employed as contrast agents in US imaging for over two decades (Cosgrove, 2006). By introducing MB into the body, they can be easily monitored using diagnostic imaging, while the application of high-intensity US pulses at the target location enables cargo delivery, minimizing negative side effects. Under ultrasound stimulation, MB promote drug convection into surrounding tissues and enhance cellular membrane permeability through sonoporation (Lentacker et al., 2014). This combination improves both the distribution of the drug within the target site and its uptake by cells (Bazan-Peregrino et al., 2012). Horsley et al. developed gas-filled lipid MB decorated with liposomes containing gentamicin and fluorescent markers for the treatment of urinary tract infections (UTIs) using a human urothelial organoid model (Horsley et al., 2019). Through US exposure (1.1 MHz, 2.5 MPa, 5,500 cycles at 20 ms pulse duration) for 20 s, MB demonstrated greater delivery effectiveness compared to the control group and was twice as effective as liposomes without MB. Importantly, no cell damage was observed. Another study by Noboru et al. demonstrated the delivery of pirarubicin into dogs’ bladders using cavitating MB induced by US (Sasaki et al., 2022). The combination of US and MB resulted in higher pirarubicin concentrations in the sonicated area compared to the nonsonicated area, while plasma concentrations of pirarubicin did not increase, and no urothelial damage was observed. In our study, we utilized US-triggered MB cavitation to deliver BTX-A intravesically and investigated the improved urodynamic manifestations and target protein cleavage of SNAP-25 in rats with AA-induced hyperactive bladder. Furthermore, the US-MB-BTX-A system demonstrated compatibility with the urothelium.

Cavitation interaction with cells or tissues induces reversible perforation of the plasma membrane, opening interendothelial junctions and stimulating endocytosis (Qin et al., 2018). These bioeffects overcome barriers and facilitate the delivery of impermeable compounds to the targeted region. Sonoporation experiments have successfully delivered small molecules, macromolecules, and nanoparticles (Deprez et al., 2021). In our study, we validated the successful delivery of BTX-A into bladder tissue by assessing the expression of cleaved SNAP-25 and employing ultrasound-induced microbubbles cavitation as an adjunct to BTX-A treatment, effectively mitigating AA-induced overactivity in cystometry. However, the direct evidence of the concentration of BTX-A in bladder tissue or serum is lacking. Thorough investigation is required to assess the safety considerations and long-term effects of ultrasound-mediated drug delivery to ensure overall safety and efficacy of sonoporation techniques. To further investigate the drug delivery process, the use of microdialysis technique is proposed to detect the concentration of botulinum toxin A in bladder and serum. Microdialysis is a minimally invasive sampling technique that allows continuous monitoring of interstitial fluid, providing real-time information on drug concentration changes in specific tissues (Kreilgaard, 2002; Gu et al., 2019). By employing microdialysis, accurate data on the actual tissue and serum concentration changes of botulinum toxin A in a rat model can be obtained, which is crucial for understanding its local distribution, potential therapeutic effects, and systemic side effects.

The cellular effects induced by cavitation are characterized by complex and interconnected mechanisms, including calcium homeostasis, pore resealing, endocytosis, and cytoskeleton organization (Deprez et al., 2021). The increased permeability resulting from sonoporation, interendothelial gap formation, and endocytosis is temporary and reversible, typically lasting from a few seconds to a few hours (Hynynen, 2008; Sheikov et al., 2008). The effect of cavitation for drug delivery relies not only on cavitation activities, which are influenced by ultrasound parameters, microbubbles properties, and the surrounding environment, but also on biological regulatory mechanisms that are essential for membrane resealing, junction restoration, and physiological recovery (Wu and Nyborg, 2008; Kooiman et al., 2014). However, the tracking of junction opening and recovery presents challenges due to limited technologies available for detecting microbubbles activities and biological responses within the vasculature (Qin et al., 2018). The duration and extent of junction opening vary depending on the ultrasound parameters and microbubbles characteristics utilized in different studies, thereby making it difficult to precisely observe the timing and degree of opening. In our study, we confirmed the transient and reversible nature of the cavitation effect induced by ultrasound microbubbles by observing the intact and continuous distribution of tight junction proteins 1 day after ultrasound treatment. Further research is required to gain a comprehensive understanding of these cellular mechanisms and pathways.

Identifying the protein target of BTX-A is crucial for understanding its localization in the bladder. Previous studies have provided conflicting data on the expression of SNAP-25 in the urinary bladder and its specific cell types (Born et al., 2003; Chuang et al., 2009; Coelho et al., 2010; Hanna-Mitchell et al., 2015; Wankel et al., 2016). Some studies have reported the presence of SNAP-25 in human and rat primary urothelial cells, while others failed to detect it in rat and mouse urothelium (Born et al., 2003; Hanna-Mitchell et al., 2015). Additionally, SNAP-25 has been observed in suburothelial nerve fibers in bladder samples using immunofluorescence (Chuang et al., 2009; Coelho et al., 2010). In our study, we found that SNAP-25 is widely distributed throughout the urothelium and suburothelial nerve fibers, while SNAP-23 is predominantly expressed in the mucosal layer. The expressions of SNAP-23 were not affected by US + MB + BTX-A pretreatment, but the levels of cleaved SNAP-25 were higher in the US + MB + BTX-A pretreated group compared to the US + MB- and BTX-A-pretreated groups. Although BTX-A has a preference for cleaving SNAP-25, it can also cleave murine SNAP-23 at high concentrations (Vaidyanathan et al., 1999; Banerjee et al., 2001). In our study, SNAP-23 expression remained unchanged between groups, in contrast to a previous *ex vivo* study by Hanna-Mitchell et al. which reported a decrease in SNAP-23 staining in rat urothelial cells after BTX-A treatment (Hanna-Mitchell et al., 2015). This difference may be attributed to the temporary turnover of umbrella cells after the application of US + MB.

Furthermore, the measurement of calcitonin gene-related peptide (CGRP) levels provides valuable insights into the underlying mechanism of action. It is believed that bladder epithelium and sensory axons participate in afferent transmission mechanisms that modulate micturition, particularly in cases of bladder inflammation and hypersensitivity. BTX-A may reduce the release of nerve growth factor, CGRP, substance P, adenosine triphosphate, and other neurotransmitters (Chuang et al., 2004; Apostolidis et al., 2005; Atiemo et al., 2005; Liu et al., 2009). After BTX-A was injected into the bladder, it inhibited CGRP release from afferent nerve terminals and provided an analgesic effect without interfering with voiding. In our study, we observed a significant increase in CGRP levels when BTX-A was administered in conjunction with ultrasound cavitation, suggesting an inhibitory effect on neurotransmitter release. These findings support the hypothesis that the combined approach of BTX-A and ultrasound cavitation modulates neurochemical signaling pathways.

The study represents the first report of BTX-A intravesical delivery facilitated by US-triggered MB cavitation. However, it is important to acknowledge the limitations of the study: 1) The mechanism of action of BTX-A in affecting sensory impulses mediated by urothelial or submucosal nerve fibers is not fully understood, despite indications of its effectiveness from CMG and molecular biology studies. 2) The precise penetration depth and site of action of BTX-A have not been directly determined. Modifications to the structure of BTX-A through fluorescence-based techniques may offer potential insights in this regard. 3) The animal models used in this study, which involved AA-induced bladder hyperactivity, may only represent a specific aspect of the pathogenesis of OAB. Therefore, caution should be exercised when extrapolating the conclusions to human studies that encompass the multifactorial and complex nature of OAB. 4) Although no leakage of toxin or systemic side effects were observed, the safety concerns associated with the use of US-triggered MB cavitation in humans, as well as the establishment of dose-effect relationships, should be thoroughly considered before its clinical application. Addressing these limitations will be important for further understanding the efficacy, mechanisms, and safety profile of BTX-A intravesical delivery facilitated by US-triggered MB cavitation.

## 5 Conclusion

In conclusion, our study demonstrates that the combination of bladder instillation of BTX-A with US-triggered MB cavitation effectively facilitates the delivery of BTX-A and prevents AA-induced bladder hyperactivity. These findings provide support for the use of US-triggered MB cavitation as an efficient method for delivering BTX-A. This approach offers a potential injection-free alternative for the treatment of overactive bladder by utilizing intravesical administration of BTX-A with the assistance of US-triggered MB cavitation.

## Data Availability

The original contributions presented in the study are included in the article/Supplementary Material, further inquiries can be directed to the corresponding author.
