# Supplementary material for: Ultrasound-assisted intravesical botulinum toxin A delivery attenuates acetic acid—induced bladder hyperactivity in rats
Source: Front Pharmacol. 2023 Jul 24;14:1214145. doi: 10.3389/fphar.2023.1214145 (PMC10406439; doi:10.3389/fphar.2023.1214145)
Supplement: Supplementary file 1 [file Table1.docx]

Supplementary Material


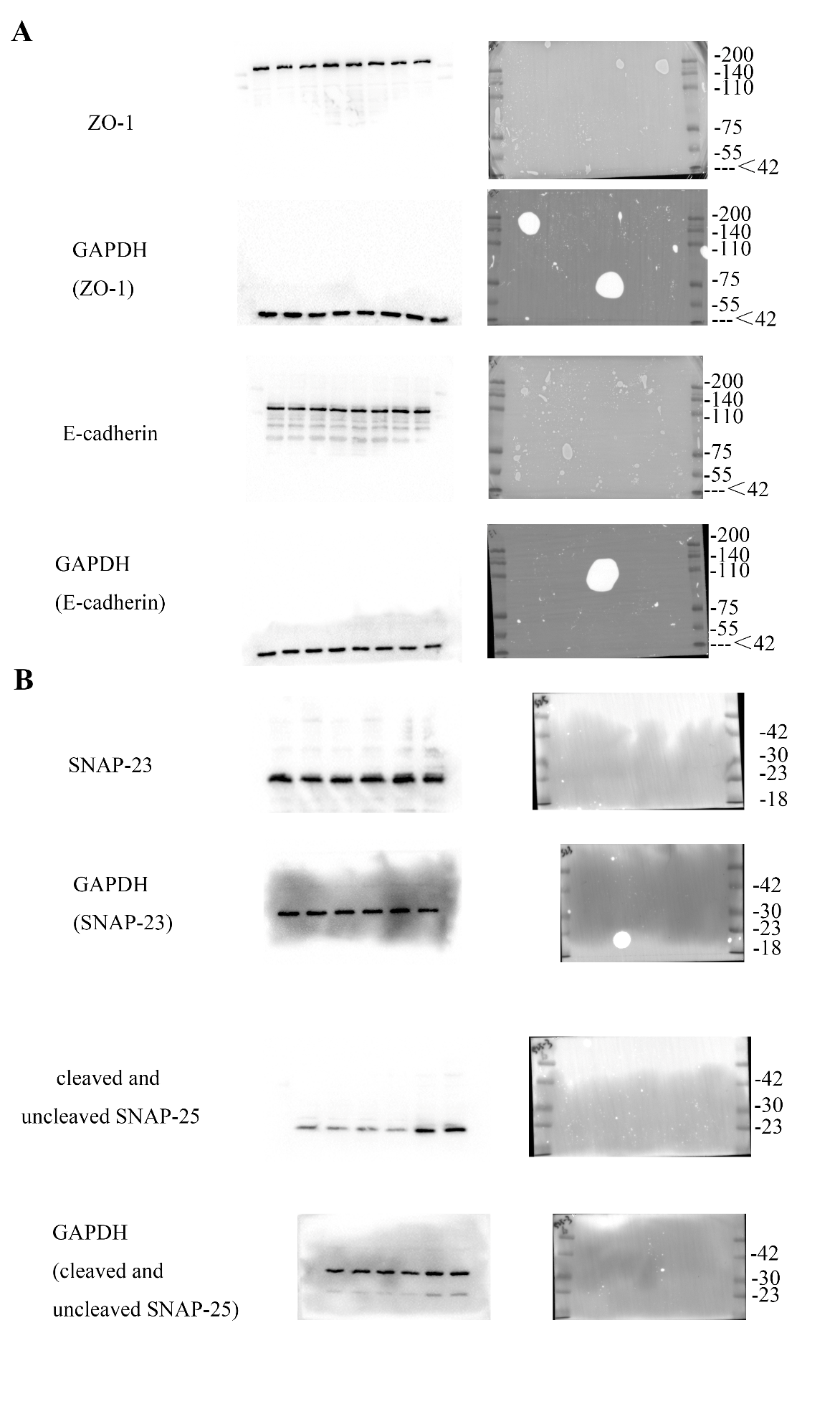


**Supplementary Figure 1.** Uncropped Western blot images. (**A**) Uncropped Western blot images from the blots shown in Figure 2. The red boxes outline the areas presented in Figure 2C. (**B**) Uncropped Western blot images from the blots shown in Figure 4. The red boxes outline the areas presented in Figure 4A. The control image of GAPDH (SNAP-23) is re-used for illustrative purposes.
